# Just Swap Out of Negative Vibes? Rumination and Inhibition Deficits in Major Depressive Disorder: Data from Event-Related Potentials Studies

**DOI:** 10.3389/fpsyg.2016.01019

**Published:** 2016-07-28

**Authors:** Aurore Monnart, Charles Kornreich, Paul Verbanck, Salvatore Campanella

**Affiliations:** Laboratoire de Psychologie Médicale et d’Addictologie, ULB Neuroscience Institute, CHU Brugmann-Université Libre de Bruxelles, BrusselsBelgium

**Keywords:** Major Depressive Disorder, MDD, inhibition, rumination, event-related potentials, emotion-regulation strategy

## Abstract

Major depression is a serious disorder of impaired emotion regulation. Emotion hyperactivity leads to excessive negative ruminations that daily hijack the patient’s mental life, impacting their mood. Evidence from past researches suggest that depressive patients present several cognitive impairments in attention and working memory, leading to a more acute selective attention for negative stimuli and a greater accessibility of negative memories. Recently, is has been proposed that impaired inhibitory functioning with regard to emotional information processing might be one of the mechanisms of ruminations linking memory, attention and depression. It seems that inhibition deficit is present at both *the input level* (i.e., the ability to reduce the interference from emotional distracters) and *the higher level* (i.e., the ability to direct the attention away from emotional material that has already been processed) of emotional information processing. Event-related potentials (ERP) have widely been used to study inhibition in adults suffering from various psychopathological states. In particular, depressive disorder has been linked to ERPs modulations, at early as well as at latter stages of the information-processing stream, when processing affective material. For instance, deficits in inhibiting negative information have been indexed by changes in the parameters (amplitudes and latencies) of early P2, P1 and N1 components while other ERP studies have shown an ability to differentiate depressed patients from normal controls based upon response inhibition difficulties in go-nogo tasks, indexed by later NoGo P3 differences. In this review, we will focus on results of ERP studies investigating inhibition and its interaction with emotional related cue processing in depressive populations. Implications for future research and theoretical perspectives will be discussed within the framework of current models of depressive disorder, based upon the hypothesis that negative ruminations are at the center of depression processes.

## Negative Vibes: Disturbances in Emotional Processing

Depressed patients seem to daily experience sustained negative affect and a persistent reduction in positive affect that impact their thoughts, behavior, mood and physical health. Importantly, those ones cannot just swap out of negative vibes. Current pharmacological and psychological treatments are quite effective in reducing depressive symptoms at short term, but relapse rates remain very high (Kessler et al., 2005). Considering that Major Depressive Disorder (MDD) affects 350 million people and is the leading cause of disability worldwide (World Health Organization [WHO], 2015), an important research challenge should be the identification of contributing factors to the development, the maintenance and the recurrence of the disease. A disrupted emotional processing has been found in depressive patients that may constitute a causal factor in the development or maintenance of clinical depression (Demenescu et al., 2010). In point of fact, healthy emotional processing has been associated with good health, relationships, academic success and work performances (John and Gross, 2004), whereas emotional dysfunctions has been related to poor social outcomes and is considered as a main causal factor in various pathological conditions such as aggressiveness, addiction, risk-taking behaviors, anxiety and depression (Davidson, 2000). It’s then worthwhile to delineate bias at the level of emotional processing in Major Depression.

### Impaired Emotional Processing in Major Depression

Major Depressive Disorder is a serious disorder of impaired emotional functioning. Behavioral studies investigating the emotional processing stream – which, ranges from an interpretation of the stimulus to the preparation of an appropriate behavioral response (Green and Leitman, 2008) – have shown that depressed patients display difficulties in the perception, the recognition and/or the regulation of emotions. In other words, compared to healthy controls, depressive individuals exhibit a disrupted emotional processing, indexed by lower performance and/or delayed response latencies (Delle-Vigne et al., 2015). For instance, emotion perception and recognition abilities have often been studied through the recognition of emotional facial expressions (EFEs) (Delle-Vigne et al., 2015). Intensity judgment task in which participants have to identify either regular EFE stimuli, either morphed stimuli, have widely been used in order to assess the ability to recognize, judge and categorize emotions. In tasks using morphed EFE stimuli, controls and depressed participants have been confronted to faces, appearing on a screen and slowly evolving from neutral to full emotional intensity. Patients were required to freeze an exhibited face and to select the best fitted emotion in a list. Such studies have shown that, compared to healthy individuals, depressed patients were less accurate in decoding anger (Mendlewicz et al., 2005), required greater intensity of emotion in order to correctly identify happy faces, but required less intensity of emotion in order to correctly identify sad and angry faces (Joorman and Gotlib, 2006), displayed longer reaction times to correctly identify sad faces than happy ones (Gollan et al., 2008), and were performing better in recognizing sad faces than in recognizing any other emotions or subtle emotional intensity (Gollan et al., 2010).

However, perception and recognition are not the only impaired faculties in the emotional functioning in depressed individuals. A deficit in emotion regulation - the processes that influence when and how emotions are experienced (Gross and Thompson, 2007; cited in Hajcak et al., 2010) – has widely been cited as a central causal factor in major depression. Indeed, it has been suggested that the possible primary dysfunction in depression not only resides in the low mood state itself, but in the brain’s inability to appropriately regulate that state (Holtzheimer and Mayberg, 2011). In point of fact, people daily experience negative events without encountering prolonged negative affect (Teasdale, 1988). Yet, something is certainly happening differently for depression-vulnerable people compared to non-vulnerable people as MDD patients are much more affected by negative experiences and are to some extend “looking on the dark side” (Hertel et al., 2014). Apparently, the difference could lie in the use of specific emotion regulation strategies not allowing patients to repair their mood once they have experienced sadness or other negative emotions (Teasdale, 1988). Indeed, some findings suggest that more frequent use of certain strategies (e.g., expressive suppressions, thought suppression, rumination, catastrophizing) and less frequent use of other strategies (e.g., reappraisal, self-disclosure) are related to levels of depression (Gross and John, 2003; Campbell-Sills et al., 2006; Garnefski and Kraaij, 2006, 2007). Most studies investigating emotion-regulation strategies in MDD have focused on rumination, which is, up to now, considered as a main causal factor of relapse in the disease (Spasojević and Alloy, 2001).

Moreover, brain imaging studies, such as functional magnetic resonance imaging (fMRI) researches, have been conducted in order to highlight the defective brain circuitry in depression that further support the idea of an impaired emotional processing in Major Depressive Disorder. Various neuropathological and neurochemical abnormalities have been found in depressive patients within the neural systems that modulate emotional behavior (Drevets et al., 2008). A particularly modified functional activity in regions involved in depression’s symptoms was widely observed. Commonly, MDD patients disclose a hyper-activated amygdala region, mainly due to a hypo-activation of prefrontal region (Dannlowski et al., 2005).

Importantly, researchers have begun to explore the neural correlates of emotion regulation strategies (Ochsner and Gross, 2005). They observed that processes, aiming to regulate an emotional state, seem to rely on a similar network of neural activation indicating a diminished emotional reactivity related to a diminished activation of the amygdala, and an increased cognitive control related to an increased activation in areas of the prefrontal cortex (Hajcak et al., 2010). For example, reappraisal has been related to an increased activation in areas of the lateral and medial prefrontal cortex and decreased activation of the amygdala (Oschner et al., 2002). When these regions miscommunicate, a hampering of the cognitive processing of emotions has been observed (Mériau et al., 2006), and it has especially been suggested that it might subtend excessive elaboration and/or rumination on negative information (Koster et al., 2011).

### Rumination in Major Depression

Rumination – a style of information processing defined by the process of recurrent thoughts and ideas (Nolen-Hoeksema, 1991) – is a prevalent trait in MDD. Indeed, depressive patients regularly engage themselves in vicious cycles of ruminative thinking focused on their symptoms, their causes and implications (Nolen-Hoeksema, 1991). Two subtypes of this process have been identified. On the one hand, reflective pondering or reappraisal, which is a solution-focused and depression-alleviating behavior. On the other hand, brooding, which consists in harmful negative interpretations and self-criticism (Treynor et al., 2003). MDD patients would mainly use the brooding subtype of rumination process that theorists consider as a particularly detrimental emotion-regulation strategy mainly increasing the hallmarks symptoms of depression (i.e., sustained negative affect and a persistent reduction in positive affect) (for a meta-analysis, see Aldao et al., 2010). Besides, it has been shown to delay recovery from negative mood and has been associated to a heightened vulnerability for the development and maintenance of depression, to higher levels of depressive symptoms, to longer and more severe episodes and might even be a mediator for the gender difference in depressive symptoms (Nolen-Hoeksema et al., 1999, 2007, 2008; Spasojević and Alloy, 2001). Indeed, approximately twice as many women as men are diagnosed with MDD (Weismann and Klerman, 1977; Kuehner, 2003) and studies using self-reported scales have shown that women had higher levels of depressed mood than men (Jorm, 1987; Nolen-Hoeksema, 1987; Kessler, 2006; cited by Leach et al., 2008). Previous studies have shown that women are more likely than men to ruminate about negative experiences or thought processes, resulting in higher levels and longer episodes of depression (Nolen-Hoeksema, 1987; Butler and Nolen-Hoeksema, 1994).

## A Cognitive Perspective for Emotion Regulation Deficits and Rumination in Depression

It’s likely that there are a number of factors that affect emotion regulation in depression. Notably, cognitive models suggest that cognitions play a crucial role in emotion regulation processing (for a review, see Mathews and MacLeod, 2005). Actually, if former theoretical models of cognitive vulnerability for depression have focused on investigating the negative content of depressogenic cognitions (for a review, see Abramson et al., 2002), recent studies have highlighted the importance of underlying cognitive processes potentially related to the sustained negative affect and impaired emotion-regulation that characterizes MDD (Joormann and D’Avanzato, 2010). Bias across several stages of emotional information-processing stream have been found to influence the etiology and maintenance of depression (for a review, see Mathews and MacLeod, 2005) and investigators have established causal connections from the cognitive impairments to features of emotional disorders (for a review, see Hertel and Mathews, 2011). It has especially been proposed that deficits in attention and working memory make negative content more accessible to depressed individuals. Indeed, negative mood has been found to be more frequently related to negative attention bias toward emotional information (Koster et al., 2005; De Raedt and Koster, 2010) and to greater accessibility of negative memories (Taylor and John, 2004; for a review, see Matt et al., 1992).

In this vein, some specific cognitive deficits have been identified as potentially leading depressed people to engage themselves in ruminative processes (Nolen-Hoeksema et al., 2008). Recent data suggests that emotional regulation processes depend on a variety of top–down strategies that includes cognitive control. For example, researchers have explored the neurophysiological correlates of various emotion regulation strategies (cfr. below for the usefulness of neurophysiological/ERPs measurements of cognitive processes), and have pointed out that emotion regulation combines both automatic and more controlled cognitive processes (Hajcak et al., 2010). Indeed, they explored the effects of various emotion regulation strategies on automatic and on later and more controlled event-related potentials (ERPs) components, such as P300 and Late Positive Potential (LPP), and found quantitative differences on the LPP’s parameters (amplitude and latencies) (Hajcak and Nieuwenhuis, 2006; Moser et al., 2006; MacNamara et al., 2009). For instance, Foti and Hajcak (2008) showed that the LPP to unpleasant pictures is reduced when a more neutral interpretation of the picture is given. They suggested that the reduced LPP might therefore reflect reduced emotional responses due to emotion regulation instructions, probably resulting from shifts in meaning and/or from the recruitment of prefrontal cortical resources associated with effective cognitive control (Ochsner and Gross, 2005).

Thus, cognitive control seems to play a major role in the use of emotion regulation strategies. Importantly, deficits in cognitive control such as inhibition, working memory updating and set shifting (Whitmer and Gotlib, 2013), and in perseverative behavior and thinking have widely been observed in ruminators samples. Among them, impaired inhibition has been identified as a potentially main causal factor in rumination (Linville, 1996; Hertel, 2004; Joormann, 2004; Cohen et al., 2014), which could provide an important link between memory and attention deficits, and depression (Joormann et al., 2007).

## Inhibition Deficits in Major Depression

### Impaired Cognitive Inhibition as a Main Mechanism of Rumination

Inhibition – pivot of cognitive control – is not a unitary construct but instead, involves several components such as response inhibition, cognitive inhibition and neural inhibition. Among them, cognitive inhibition refers to an active process that tempers unwanted external and/or internal stimuli that compete for processing resources in the context of limited capacity system (Hasher and Zacks, 1988). Dealing with negative emotional situations and negative mood states then requires effective cognitive inhibition. That is, it allows people to stop the processing of an activated negative material in working memory and to reorient their attention to other aspects of the situation (Joormann, 2010). Basically, inhibition operates at different levels of the information-processing stream (Hasher and Zacks, 1988) as it can both allow people to reduce the interference from emotional distracters (input level) and to direct attention away from emotional material that has already been processed and needs to be removed from working memory (higher level).

### Exploring the Inhibiting Brain Through Behavioral and ERPs Studies

Inhibition of emotional content may be explored through several approaches. To date, it looks like they have been more behavioral researches on inhibition in depressed samples than cerebral mechanisms studies.

In order to assess inhibitory functioning, either at the input level or at the higher level of emotional processing stream, cognitive psychologists have investigated distracter inhibition, interference inhibition and inhibition of return (IOR) in behavioral tasks that required participants to ignore emotional (positive or negative) material to response to a target stimulus such as the emotional Stroop Task (i.e., interference inhibition; Yovel and Mineka, 2005), the negative affective priming task (NAP) (i.e., distracter inhibition; Joormann, 2004; Goeleven et al., 2006), the cue-target task (i.e., inhibition of return; Dai and Feng, 2009) and the Go-NoGo task (Erickson et al., 2005). Meanwhile, neuroscience has completed behavioral measures by elucidating neural correlates associated with impaired information processing in various psychiatric diseases. Especially, the development of brain imaging techniques, such as fMRI, provided the possibility to explore brain regions involved in emotional processes and how they fail to interact in depression (Dannlowski et al., 2005; Mériau et al., 2006; Kerestes et al., 2012). For example, recent neuroimaging data, with a good spatial resolution, has allowed researchers to observe impairments at the neural level and to further support previous behavioral results (Rogers et al., 2004). The idea of exploring the neural correlates associated with depression-related impaired inhibition over emotional stimuli is then encouraging. However, fMRI suffers from poor temporal resolution. Cognitive functions, such as inhibition, require various steps and cognitive stages (serially or in parallel) to give rise to a normal performance. The origin of a behavioral impairment may thus arise from the alteration of a particular cognitive stage differently situated along the information-processing stream. The possibility to access dynamic temporal information should then be promoted and techniques effective enough to explore real time brain activity in the range of milliseconds should be used. A possible way to obtain a complete overview of the information processing across time is to use ERPs as they reflect changes in brain activity at early and late latencies. Actually, ERPs consist in several components, each one characterized by two main parameters: amplitude and latency. It’s believed that the amplitude represents the degree of brain activation during a cognitive task, reflecting the attentional resources occupied during the task, and the latency represents the speed at which the stimulus is perceived, reflecting the time needed to discriminate the stimuli (Olofsson et al., 2008). Changes in the parameters (amplitudes and latencies) of some ERP’s components, considered as an electrophysiological index of cognitive functioning (Miller, 1996), may so index particular cognitive impairments (Rugg and Coles, 1995).

### Paradigms

#### The Emotional Stroop Task

**FIGURE 1:**
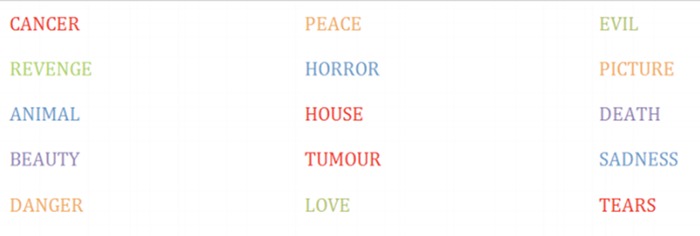
**Illustration of an Emotional Stroop Task.** Participants are asked to name the ink color as fast as possible for each presented words.

In the emotional Stroop task, participants are confronted to emotional words written in different colors (see **Figure 1** below for an illustration). They are required to name colors of positive, neutral and/or negative words. Researchers then examine time responses for each emotional condition, reflecting different mechanisms of interference, which include interference inhibition. Note that this task, traditionally used in neuropsychological studies on inhibition, does not allow distinguishing between active selection of task relevant material and active inhibition of task-irrelevant (emotional) material (Hasher and Zacks, 1988).

#### The Negative Affective Priming Task

**FIGURE 2:**
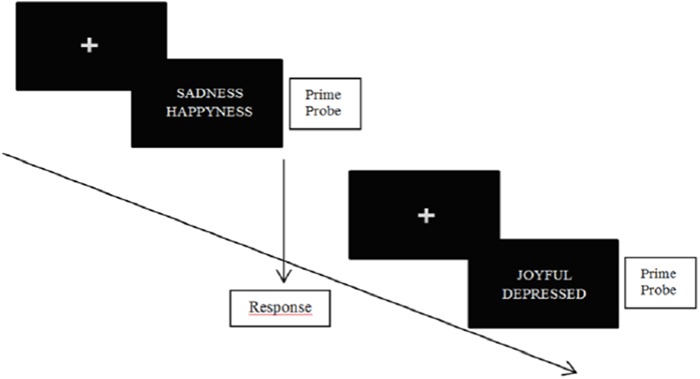
**Illustration of NAP task.** Participants are asked to specify the valence of the probe stimulus as fast as possible.

In classic NAP tasks, subjects are simultaneously confronted with two emotional stimuli (e.g., emotional words and/or emotional faces) (see **Figure 2** below for an illustration). One of those stimuli consists in a probe trial, and the other consists in a prime trial. Subjects have to specify the valence of the probe trial as fast as possible. Accordingly, the time necessary to respond depends on the prime trial. Indeed, the probe trial is processed faster when the prime is valence-congruent, while slower when the prime is valence-incongruent. On the following trial, the previous prime may become the new probe trial (or not). In this special case, priming occurs because the prime triggers other information of the same valence (Fazio et al., 1986). The power of cognitive inhibition on the first trial is indexed by the response latency for the new probe trial (Kircanski et al., 2012). A modified version of the negative priming paradigm (Joormann, 2004) allows examining both the possibility of enhanced facilitation and impaired inhibition at the input level in a single design.

#### The Cue-Target Task:

**FIGURE 3:**
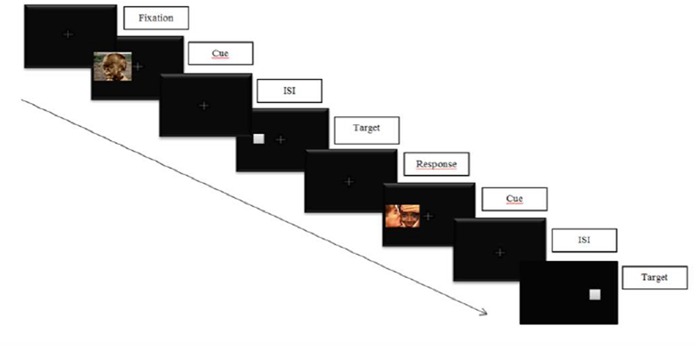
**Illustration of an emotional cue-target task.** Participants are required to respond to the target location while inhibiting informations on cue’s location.

In a classical cue-target task, participants are required to response to the location of a target appearing after a cue (see **Figure 3** below for an illustration). Two conditions can occur: (1) a valid cue condition in which the target appears at the same location as the cue, and (2) an invalid cue condition in which the target appears at a different location as the cue. Cue validity is inferred if the reaction times (RTs) in the valid cue condition are significantly shorter than those in the invalid cue condition. On the contrary, an IOR effect is obtained if the RTs in the valid cue condition are not significantly shorter than those in the invalid cue condition.

#### The Go-NoGo Task:

**FIGURE 4:**
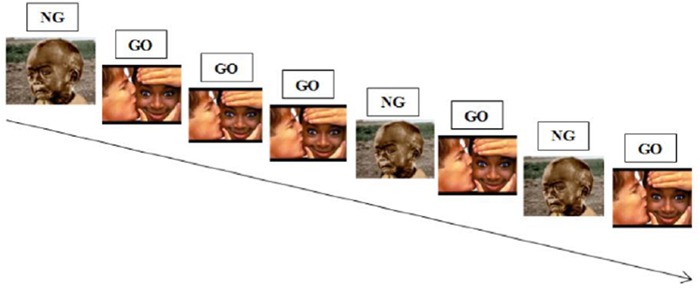
**Illustration of an emotional Go-NoGo task.** Participants are required to respond to positive stimuli while inhibiting responses to negative stimuli.

During a classic Go-NoGo task, participants have to respond as fast as possible to one stimulus (“Go” stimulus), which set up a prepotent response tendency and not to another one (“NoGo” stimulus), which require that prepotent response tendency to be inhibited (see **Figure 4** below for an illustration). In an affective Go-NoGo task, subjects are required to respond to stimuli of one valence while inhibiting responses to stimuli of the opposite valence (Erickson et al., 2005; Bermpohl et al., 2006).

### ERPs Components Reflecting Inhibition

Event-related potentials have widely been used to study neuronal processing related to inhibition in adults suffering from various psychopathological states. With regard to MDD, ERPs measured during cognitive inhibition tasks, such as the previously cited ones, revealed some modulation at early as well as at latter stages of the information-processing stream when processing affective material. Indeed, tasks looking for neurophysiological indices for impaired inhibition have respectively focused on several relevant ERPs components thought to index cognitive processes involved in inhibition. Early (P1, P2, N1 and N2) and late (P3, No-Go P3, N450, LPC) components have been explored depending on the tasks used to assess cognitive inhibition. That is, neuronal processing related to cognitive inhibition can be examined with electroencephalography (EEG) when combined with particular paradigms from used in cognitive psychology studies.

#### Early P1, P2, N1, N2

Firstly, detected around 60–140 milliseconds (ms) after stimuli onset, the P1 component consists in a positive deflection, which is thought to index the low-level features of stimuli and initial encoding for sensory information (Pierson et al., 1996; cited by Yang et al., 2011). Secondly, detected around 100–200 ms after stimuli onset, the N1 component consists in a negative deflection thought to index the attentional focus on target and a discrimination process within the focus of attention (Dai et al., 2011). Thirdly, detected around 160–210 ms after stimuli onset, the P2 component consists in a positive deflection, which is thought to index the detection of visual aspects at perceptual stages of information processing (Luck and Hillyard, 1994; cited by Yang et al., 2011). Above all, P2 with an anterior distribution on the scalp is also thought to reflect the initial difference of task-relevant stimuli from task-irrelevant stimuli. (Lindholm and Koriath, 1985; Hegerl and Juckel, 1993; cited by Yang et al., 2011). Finally, detected around 250–430 ms after stimuli onset, the N2 component consists in a negative deflection thought to index further evaluation of targets (Ritter et al., 1979; cited by Yang et al., 2011). Above all, N2 is also thought to reflect cognitive control, mismatch detection and affective experiences (Deldin et al., 2000; Folstein and Van Petten, 2008; cited by Yang et al., 2011).

#### Late P3, No-Go P3, LPC, N450

Firstly, detected around 300–600 ms after stimuli onset, the P3 component consists in a positive deflection thought to index late evaluation stage of processing and updating in working memory (Donchin and Coles, 1988; cited by Yang et al., 2011). Evaluated in Go-NoGo tasks, NoGo-P3 is thought to index cognitive inhibition function (Smith et al., 2013). Secondly, detected around 400–500 ms after stimuli onset, the N450 component consists in a negative deflection thought to index conflict processing. Importantly, it has been correlated to cognitive inhibition in the standard Stroop Task (McNeely et al., 2008). Finally, detected around 400–800 ms after stimuli onset, the Late Positive Component (LPC) consists in a positive deflection thought to index working memory updating at late evaluation stage of processing (Donchin and Coles, 1988; cited by Yao et al., 2010).

### Inhibition Disturbances in Depression

#### Behavioral Studies

From now on, behavioral studies investigating cognitive inhibition deficits in MDD, through the used of inhibitory tasks described above, have found consistent impairments both in the processing of neutral material (Moritz et al., 2002; Erickson et al., 2005; Stordal et al., 2005; Markela-Lerenc et al., 2006; Gohier et al., 2009), and in the processing of emotional information (Joormann, 2004, 2010; Goeleven et al., 2006; Frings et al., 2007; Joorman and Gotlib, 2008; Dai and Feng, 2009; Yao et al., 2010; Dai and Feng, 2011; Dai et al., 2011). In this case, they have observed that depression is associated with impaired inhibition both at the input level (i.e., the ability to reduce the interference from emotional distracters) and the higher level (i.e., the ability to direct the attention away from emotional material that has already been processed) of the emotional information processing stream (Joormann, 2004; Goeleven et al., 2006; Joorman and Gotlib, 2008). Importantly, those studies have observed diminished inhibition ability in response to negative stimuli in depressed patients compared to control samples. For example, behavioral data obtained through NAP tasks suggested that, compared with control participants, MDD patients had enhanced negative priming and less inhibition of sad faces. For example, using a NAP task, Joormann (2004) have observed an altered inhibition toward negative words in depressive patients (MDD) and in remitted depressed patients (RMD). In another NAP study using facial emotions, Goeleven et al. (2006) observed a less effective inhibition specifically toward negative information in MDD individuals compared to never depressed patients (NC) and RMD subjects. Interestingly enough, mixed results have been found considering eventual inhibition impairments in RMD patients (Joormann, 2004; Goeleven et al., 2006). Indeed, while Joormann (2004) have observed a specific impaired inhibition for negative material in formerly depressed patients as in currently depressed patients, Goeleven et al. (2006) have observed that formerly depressed individuals demonstrated impaired inhibition of both negative and positive information.

Importantly, most of those studies have suggested that the valence-specific (negative) inhibition impairment observed in MDD play a crucial role in rumination (Joormann, 2004; Joorman and Gotlib, 2006; Frings et al., 2007; De Lissnyder et al., 2010; Joormann and Gotlib, 2010; Zetsche and Joormann, 2011; Cohen et al., 2012, 2014, 2015; Daches and Mor, 2014). That is, when inhibition process malfunction, it might set the stage for ruminative responses to negative events and negative mood states due to a prolonged processing of negative, goal-irrelevant information (Koster et al., 2011). In other words, inhibitory dysfunction might reduce the control of access of negative cognitions into working memory. Given the capacity limitations of this system, it could lead to difficulties in attending to new information and reorient the attention to other aspects of the situation (Joormann and Gotlib, 2010), thereby hampering recovery from negative mood and leading to increased levels of negative affect Inhibition deficits might then link attention (Koster et al., 2005; De Raedt and Koster, 2010; Koster et al., 2011), memory (Hascher et al., 1999; cited by Lyubomirsky et al., 1998; Gohier et al., 2009) and rumination in MDD patients (Joormann, 2010). For instance, Koster et al. (2011) suggested that inefficient inhibitory functioning might be at the heart of difficulty to disengage attention away from irrelevant negative information, which contributes to rumination. Therefore, a major challenge of up-to-date research is to assess inhibitory functioning in MDD population.

#### Event-Related Potentials Studies

Only recently, studies have begun to explore the neural correlates associated with depression-related impaired inhibition over emotional stimuli with electroencephalography (EEG) during particular tasks in which participants have to inhibit some task-irrelevant information. Mounting ERP evidence indicates that depressive individuals display inhibitory dysfunction at the input and the higher level of the information processing-stream. We summarized here ERPs studies investigating inhibition in MDD.

Dai et al. (2011), interested in exploring the neural correlates of distracter inhibition ability for emotional faces in MDD patients, have used a modified emotional NAP task combined with ERPs. They have applied the task in control individuals who had never suffered from depression (NC), remitted depressed patients (RMD), and major depressive disorder patients (MDD). Their behavioral results suggested that MDD patients, compared to controls, had enhanced positive priming and less inhibition of sad faces, and those RMD patients, compared to controls, had general inhibitory deficits for all emotions faces and facilitation for sad faces. Accordingly, their neurophysiological results revealed that MDD patients displayed larger P1 and P3 amplitude for sad faces in the positive priming condition compared to both NC and RMD groups; and smaller P3 amplitude for sad faces in the negative priming condition. Those results suggest a deficient distracter inhibition and an excessive facilitation for negative stimuli in MDD patients. Interestingly, a deficient inhibition and an excessive facilitation for both positive and negative information were found in RMD patients.

In another study, Yao et al. (2010) have used a similar experimental design as they combined an affective NAP task while recording early P2 and later LPC components. At a behavioral level, they found a less effective inhibition for negative material. At a neurophysiological level, they observed an overall diminution in P2 amplitude for negative trials, and an overall diminution in LPC amplitude for both negative and positive trials. Specifically, their results also suggest that MDD patients have decreased central-parietal P2 amplitude and decreased LPC amplitude for negative material compared to controls.

Furthermore, interested in exploring the neural correlates of interference inhibition ability for emotional words in MDD patients, Dai and Feng (2011) have used a modified emotional Stroop task combined with ERPs. Comparing control individuals who had never suffered from depression (NC), subclinically depressed patients (RMD), and major depressive disorder patients (MDD), they found that MDD patients are characterized by behavioral (e.g., MDD patients have a higher interference effect for negative words than NC and RMD groups) and neurophysiological indices (e.g., diminished N1 amplitude for negative words and a diminished P1 amplitude for positive words in MDD patients compared to the other groups) for impaired attentional inhibition for negative information. Interestingly, impaired attentional inhibition for negative words was only observable in terms of neurophysiological responses in RMD patients (both RMD and MDD groups displayed enhanced N450 amplitude over the parietal regions for negative words compared to controls).

Another research has used a cue-target task to investigate the phenomenon of inhibition of return (IOR) in depressed individuals. In this study, Dai and Feng (2009) using emotional faces as cues, have applied their paradigm, combined with ERPs, on three groups: control individuals who had never suffered from depression (NC), remitted depressed patients (RMD), and major depressive disorder patients (MDD). They found that depressed patients had cue validity and a deficient IOR for negative stimuli that makes them enable to eliminate the interference of negative stimuli and might causes the development and the maintenance of depression (Dai and Feng, 2009). Interestingly, RMD participants had cue validity and a deficient IOR for both positive and negative stimuli, which, according to the authors, makes them enable to maintain emotional balance. Indeed, they observed in MDD patients larger P1 amplitude for sad cue compared to the others groups, larger P3 amplitude for sad cue than for other faces cues, smaller P3 amplitude for sad faces in the invalid cue-condition compared with the NC group, and smaller P3 amplitude for happy faces in the valid cue condition compared with the other groups.

In summary, behavioral studies have shown that MDD patients present inhibition dysfunction for negative material. Importantly, those results are mirrored in ERPs data, which shows that it modulates the earlier attention allocation stage as well as the later evaluation stage.

## Toward a Better Knowledge of Inhibition Deficits Through ERPs’

Depressed patients seem to daily experience sustained negative affect and a persistent reduction in positive affect. Several questions remain to find out why depressed patients cannot just swap out of negative vibes. Therefore a major research challenge is to identify contributing factors to the development, the maintenance and the recurrence of Major Depressive Disorder. It has been shown that the system that filters emotionally relevant information from irrelevant one is impaired in patients with Major Depressive Disorder and that this could underlie rumination by linking depression, attention and memory deficits observed in the disease. We reported here behavioral and ERPs results related to inhibition. Importantly, ERPs data mirror behavioral results showing that MDD patients present inhibition dysfunction for negative material, which modulates the earlier attention allocation stage as well as the later evaluation stage. Several theoretical and clinical implications for future developments arise from this.

Indeed, better theoretical knowledge of Major Depressive Disorder and its underlying cognitive deficits are needed. ERPs, with its good temporal resolution, seem to be a preferred technique to study cognitive impairments at a neural level. Moreover, ERPs could allow us to further deep into inhibitory processes. For instance, recent studies have suggested that diminished inhibitory control in response to a negative stimulus might in fact stem from a breakdown in both reactive and proactive cognitive control (Braver, 2012; Vanderhasselt et al., 2012, 2014). That is, Braver (2012) in the Dual Mechanisms of Control framework (DMC), states that cognitive inhibitory control consists of two complementary mechanisms in response to an imperative stimulus (e.g., conflict) that operate at different moments during conflict monitoring. On the one hand, proactive control appears early and refers to anticipatory or preparatory processes (i.e., activating and maintaining online goal-relevant information). On the other hand, reactive control appears later during conflict monitoring and acts as a correction mechanism that is activated when an ambiguous or conflict stimulus occurs (Jacoby et al., 1999). In recent studies, researchers have precisely resorted to ERPs to assess the amount of proactive and reactive control in depressed samples (Vanderhasselt et al., 2012, 2014). Furthermore, Major Depression Disorder is a heterogeneous mental illness (e.g., symptomatology, course, and treatments) (Downar et al., 2014). Subtypes of depression have been identified such as melancholia and non-melancholia and ERPs have already shown their discrimination power with regard to depression subtypes (Kemp et al., 2010). ERPs could then be considered as tools for parsing the heterogeneity of depression in ways that are intrinsically relevant for treatment selection.

In this vein, using ERPs could be of major relevance for clinicians. Firstly, future diagnosis and treatment procedures could be facilitated by the use of ERPs as they have shown in previous studies their ability to discriminate between currently depressed patients remitted patients and never depressed controls (Dai and Feng, 2009, 2011; Kemp et al., 2009; Dai et al., 2011; Delle-Vigne et al., 2015). Secondly, if traditional psychiatry has focused on behavioral symptoms rather than neurophysiological criteria to study mental disorders, their pathology, and orient their treatments, recent studies have demonstrated the possibility of using ERPs data as a potential state biomarker of various psychiatric disorders such as alcoholism (Petit et al., 2014) and depression (Kemp et al., 2009). Indeed, ERPs could be sensitive to some behaviorally invisible vulnerability. By indexing which stage of the inhibition process is impaired and revealing some behaviorally invisible vulnerabilities, ERPs could influence the choice of treatment for clinicians and therapists.

An interesting question would be whether boosting inhibition in MDD patients could lead to less rumination. For instance, cognitive training (Ditye et al., 2012) and neuromodulation techniques, such as repetitive Transcranial Magnetic Stimulation (rTMS) or transcranial Direct Current Stimulation (tDCS) (Hsu et al., 2011; Ditye et al., 2012; Juan and Muggleton, 2012; Campanella et al., 2016), have already been used to boost inhibitory functioning in normal controls and other psychopathological states. Indeed, cognitive training is an effective tool to improve a variety of cognitive functions. Regarding tDCS, it has previously been demonstrated that stimulation over the right inferior frontal gyrus (rIFG) facilitates behavioral inhibition performance and modulates its neurophysiological correlates (Campanella et al., 2016). Recently, researchers have begun to combine cognitive training and brain stimulation in order to assess the enhancing/synergic effect of those techniques. For instance, the study of Ditye et al. (2012) aimed to investigate the behavioral facilitation in the context of a learning paradigm by giving tDCS over the rIFG repetitively (i.e., 1.5 mA during 15 min) over four consecutive days of training on a behavioral inhibition task [stop signal task (SST)]. Their findings suggest that tDCS combined with cognitive training is effective for improving inhibitory functioning. In this view, the next step might be to investigate whether combining different techniques in order to boost inhibitory functioning could be of some relevance in clinical population in order to improve their pathological state.

With their potential to measure the evolution of resistance to distracting interferences, ERPs could be used as a way to assess inhibitory control, when patients are processing negative emotional stimuli, from baseline to one of those treatments’s endpoint. Furthermore, in the aim to individualize treatment based on the personal characteristics of each patient, ERPs could help to predict which patient could benefit from those treatments. That is, patients who don’t display inhibition deficits, when viewing negative emotional stimuli at baseline, should presumably not benefit from inhibition improvement treatments. Therefore ERPs combined to an inhibition task at baseline of treatment could have predictive value and be useful in the selection of patients who could benefit from it. However, further investigations are needed for neurophysiological biomarkers being regularly used in clinical psychiatry, which requires multi-guidelines to be developed for ERPs recording (Campanella and Colin, 2014).

## Conclusion

Major Depressive Disorder patients cannot just swap out of negative vibes. Based upon the hypothesis that negative ruminations are at the center of depression processes and are probably underlined by impaired cognitive inhibition, we would preconize a multi-disciplinary treatment approach of the disease including social, psychological and medical support/treatment. Among them, neuromodulation techniques, such as transcranial Direct Current Stimulation (tDCS), Mindfulness and cognitive inhibition training could enhance depressed patients’ abilities to inhibit negative ruminations. ERPs, given their high sensitivity for cognitive impairments, could play a crucial role by highlighting which impaired cognitive process should be trained in order to improve the patient’s clinical state.

## Author Contributions

All authors listed, have made substantial, direct and intellectual contribution to the work, and approved it for publication.

## Conflict of Interest Statement

The authors declare that the research was conducted in the absence of any commercial or financial relationships that could be construed as a potential conflict of interest.
